# Quality improvement of shrimp (*Litopenaeus vannamei*) during refrigerated storage by application of Maillard peptides/water‐soluble chitosan coating

**DOI:** 10.1002/fsn3.2894

**Published:** 2022-04-22

**Authors:** Yu Liu, Yanling Zhu, Yang Yang, Shiwei Hu, Wei Jiang

**Affiliations:** ^1^ Key Laboratory of Key Technical Factors in Zhejiang Seafood Health Hazards National Engineering Research Center for Marine Aquaculture Zhejiang Ocean University Zhoushan China

**Keywords:** chitosan, coating, Maillard peptides, shelf life, shrimp

## Abstract

We investigated the effect of squid Maillard peptides (SMPs) on the shelf life and quality of shrimp for 20 days. Water‐soluble chitosan coatings incorporated with SMPs (SMPs + chitosan) were applied to shrimp under chilled conditions. Untreated samples were used as control, along with samples treated with water‐soluble chitosan and SMPs alone. The pH increase was observed in all samples, as well as increased total plate count, total volatile basic nitrogen, peroxide value, and thiobarbituric acid index. However, these indexes in the SMPs + chitosan group were lower than the other three groups, which suggested SMPs + chitosan might play a role in retarding quality loss of shrimp, and there might be a combined effect between water‐soluble chitosan and SMPs. Based on hardness, springiness, and sensory evaluation, shrimp coated with SMPs + chitosan was the best preserved, with a shelf life of 16 days but only 8–12 days for other samples. The present work demonstrates the effectiveness of SMPs + chitosan, offering a promising alternative to inhibit microbial growth and lipid oxidation on shrimps during refrigerated storage.

## Graphical abstract

This study investigated the effect of chitosan coating combined with squid Maillard peptides (SMPs + chitosan) on the quality of Pacific white shrimp during iced storage. The SMPs + chitosan treatment was found to provide superior preservation of shrimp than either of the two components alone or the control. In addition, a possible synergistic effect between SMPs and chitosan, leading to inhibition of shrimp quality loss, was observed.

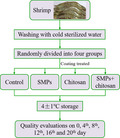

## 1 INTRODUCTION

Shrimps are rich in protein and omega‐3‐fatty acids, which are excellent food sources. However, shrimps are highly susceptible to microbiological spoilage, which leads to changes in texture, taste, and color, and the consequent low consumer acceptance (Canizales‐Rodríguez et al., 2015; Zhang et al., 2015), limiting their shelf life (Eddin & Tahergorabi, 2017). Frozen storage is one of the most important preservation methods in shrimp processing that effectively terminates microbial spoilage. However, quality deterioration still occurs during frozen storage (Arancibia et al., 2015; Okpala et al., 2014). Moreover, although frozen storage can effectively delay the physical and chemical changes of shrimp, black spot formation (melanosis) may occur after thawing (Díaz‐Tenorio et al., 2007).

In recent years, the application of natural preservatives in the food industry has gained immense interest (Sharma et al., 2021; Zheng et al., 2022). However, certain disadvantages, such as sensory properties changes, quick release of active ingredients, and interaction with other food ingredients, were observed during direct application. The coating is a good method to resolve these problems, incorporating active compounds and increasing the shelf life of seafood such as shrimp (Zhang et al., 2019, 2020). Treating shrimp with active compounds, such as cinnamaldehyde (Mu et al., 2012), seed extracts from grapefruit (Lalithapriya et al., 2017), and catechin (Nirmal & Benjakul, 2010), reportedly improve the shelf life of shrimp. Thus, the development of materials with the film‐forming ability and antimicrobial properties, which help improve shrimp safety and shelf life, has gained immense research interest (Jiang et al., 2019; Nirmal & Benjakul, 2010). Edible coatings incorporated with active compounds can delay lipid oxidation, prevent protein function loss, and improve the quality of shrimp (Arancibia et al., 2015).

Maillard peptide is formed by carbonyl compounds such as reducing sugars and peptide‐bound amino acids. In recent years, the antioxidant and antimicrobial effects of Maillard peptides have been widely studied. It has been reported that the Maillard peptides have high antioxidant properties (Liu et al., 2014) and high antibacterial activities against different strains of bacteria (Hauser et al., 2014; Song et al., 2017). We previously demonstrated that squid Maillard peptides (SMPs), generated from squid waste peptides and d‐arabinose, exhibit high antioxidant and antibacterial activities (Jiang et al., 2018). Thus, the SMPs showed a high potential usage in food preservation.

Chitosan, a polysaccharide obtained from the deacetylation of chitin, displays relatively high antioxidant and antibacterial activities. Chitosan effectively improves the quality and shelf life of seafood (Wang et al., 2015; Wu, 2014; Zamani et al., 2022). However, its application in specific fields is limited as it is insoluble in water at neutral pH, which is an essential requirement in food, health, and agricultural industries (Chouljenko et al., 2016; Dayarian et al., 2014). Carboxymethyl chitosan is a water‐soluble derivative of chitosan. It exhibits relatively high antioxidant and antibacterial activities and is used to produce edible films or coatings. To the best of our knowledge, the present study is the first to investigate the impact of water‐soluble chitosan coating in combination with Maillard peptide on the shelf life and quality of shrimp. Thus, this study aimed to evaluate the effect of a chitosan coating combined with Maillard peptide (SMPs + chitosan) on the quality of Pacific white shrimp under chilled conditions.

## 2 MATERIALS AND METHODS

### 2.1 Schematic overview of the study

**FIGURE 1:**
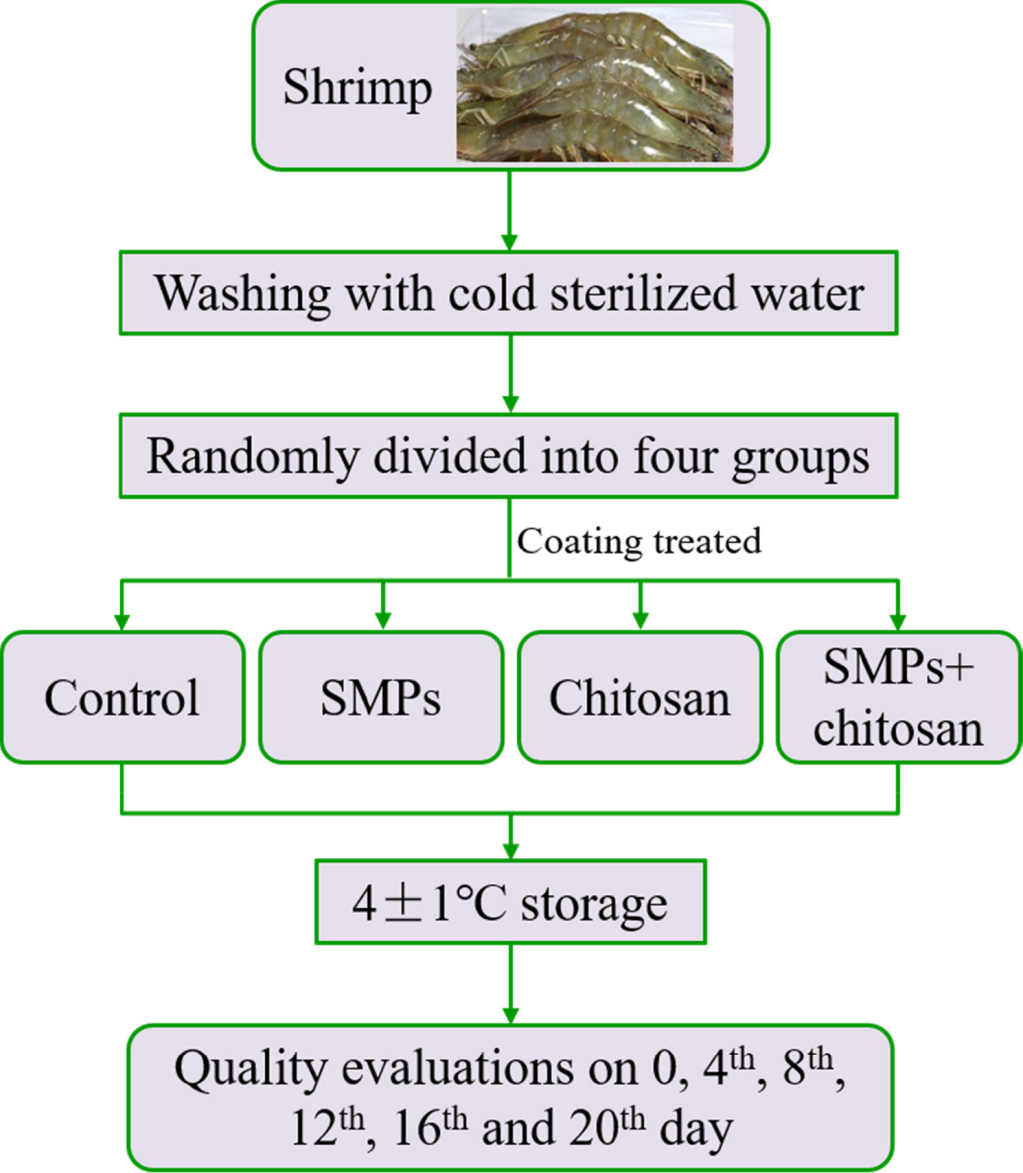
A schematic overview of the experimental study. Control: shrimp without coated; SMPs: shrimp coated with SMPs; chitosan: shrimp coated with chitosan; SMPs + chitosan: shrimp coated with SMPs and chitosan

Figure 1 illustrates a schematic overview of the study describing the entire experimental process, from sampling to storage and analysis.

### 2.2 Chemicals

Squid by‐products (raw materials), consisting of a visceral ink sac, were collected directly from the pilot plant transformation line hygienically and stored at −20°C for further use. d‐Rabinose and chitosan were purchased from Solarbio. Pepsin was obtained from Sigma‐Aldrich. Ultrapure water was prepared using a Milli‐Q (Millipore). The other chemicals used were of analytical grade and were obtained from Sinopharm Chemical Reagent Co. Ltd.

### 2.3 Preparation of coating solution, coating, and storage

The SMPs used in this study were generated from squid wastes peptides and d‐arabinose. To prepare the squid wastes peptides, 100 g of squid wastes were homogenized in 100 ml of deionized H_2_O and heated to 85°C to inactivate the endogenous enzymes for 10 min. The pH of the homogenate was adjusted to 3.0 using HCl after cooling to 35°C. The proteins were digested using pepsin (0.04 U/mg of protein) for 1 h. Following hydrolysis, the mixture was heated at 85°C for 10 min to terminate the reaction. After that, the hydrolysates were centrifuged at 7155 *g* for 10 min, and the supernatants were collected. The final fractions (soluble peptides) were freeze‐dried and stored at 4°C.

The SMPs were prepared as follows: 100 ml of squid wastes peptides (solid content 100 mg/ml) and 100 ml of d‐arabinose (150 mg/ml) were mixed. The pH of the mixture was adjusted to 12.0 using NaOH. After that, the mixture was heated for 90 min at 100°C. The products were cooled immediately to stop the reaction. The products were lyophilized and stored at 4°C for further use.

Shrimps (*Litopenaeus vannamei*) were collected from a fish market in Zhoushan, Zhejiang, China. They were transported alive to the laboratory in a large cooler box in <20 min. The average weight of a shrimp was 18 ± 2 g. Upon arrival, the samples were washed with potable cold water and stored in an icebox. Next, the samples were randomly assigned into four groups: control (uncoated), SMPs, chitosan, and SMPs + chitosan groups. The shrimps in the SMPs, chitosan, and SMPs + chitosan groups were immersed in the SMPs solution (0.5 mg/ml), 1% chitosan solution, and SMPs solution + chitosan solution (the final concentration of SMPs was 0.5 mg/ml), respectively, at a shrimp/solution ratio of 1:2 (w/v) at 4°C for 30 min. The shrimps were drip‐dried at 4°C for 5 min. The control group was immersed in deionized H_2_O at 4°C for 30 min and then dried at 4°C for 5 min. All samples were placed in plastic bags and stored in a refrigerator (4°C ± 1°C) for 20 days. Quality attributes were evaluated every 4 days.

### 2.4 Chemical analyses

#### 2.4.1 pH

The samples were homogenized in 10 volumes (w/v) of chilled distilled water for 10 s and incubated for 30 min. The homogenate was centrifuged at 2795 *g* for 10 min at 4°C. The pH of the supernatant was measured using a digital pH meter (Echeverría et al., 2018).

#### 2.4.2 Thiobarbituric acid (TBA) index

The TBA index was evaluated as described previously (Tarladgis et al., 1960). TBA values were expressed as milligrams of malonaldehyde equivalents per kilogram of the sample (mg MAD/kg).

#### 2.4.3 Peroxide value (PV)

PV was determined using a previously described method (Pereira et al., 2010). Water was used instead of samples as a reagent blank. PV was expressed as milliequivalents of peroxide oxygen per kilogram of the sample (meq peroxide/kg sample).

#### 2.4.4 Total volatile‐based nitrogen (TVB‐N)

TVB‐N analyses were performed as described previously (Djamal et al., 2018). The TVB‐N values were expressed as mg N/100 g sample.

### 2.5 Microbiological analysis

The samples were homogenized with nine times the volume of sterile water (w/v) for 1 min. Next, the sample was diluted in sterile water via a ten‐fold dilution gradient. The samples were plated via spreading on nutrient agar and incubated at 37°C for 24 h. Microbial colonies were calculated as log CFU/g of the sample (Yuan et al., 2016).

### 2.6 Texture properties

A texture analyzer (TMS‐Pilot, FTC), used to evaluate the texture (hardness) of tuna samples, was utilized according to a previously described method (Zhang et al., 2015). The texture profile analysis (TPA) was performed as follows: sample deformation, 40%; constant test speed, 1.0 mm/s; hold time between cycles, 5 s; and trigger force, 1 N. Hardness was defined as the maximum force (N) that occurs at the first compression cycle.

### 2.7 Sensory evaluation

Sensory analysis was conducted by eight trained panelists. The overall quality was rated based on the color, odor, and texture on a 1–9 scale (Table 1). The maximum shelf life was defined based on the day when the score was ≥4 (Ojagh et al., 2010).

**TABLE 1:** Sensory scores of shrimps during storage (4 ± 1℃) for 20 days

Sensory scores	0	4	8	12	16	20
Control	9.00 ± 0^d^	7.12 ± 0.57^c,A^	4.99 ± 0.75^b,A^	2.64 ± 0.45^a,A^	NA	NA
SMPs	9.00 ± 0^e^	8.54 ± 0.25^e,B^	7.68 ± 0.44^d,B^	4.69 ± 0.66^c,B^	3.81 ± 0.41^b,A^	1.48 ± 0.14^a,A^
Chitosan	9.00 ± 0^e^	8.49 ± 0.76^e,B^	7.33 ± 0.63^d,B^	4.99 ± 0.51^c,B^	3.64 ± 0.62^b,A^	1.36 ± 0.18^a,A^
SMPs + chitosan	9.00 ± 0^d^	8.86 ± 0.74^d,B^	8.02 ± 0.77^d,B^	6.11 ± 0.55^c,C^	4.53 ± 0.51^b,B^	2.57 ± 0.16^a,B^

Note

Control: shrimp without coated; SMPs: shrimp coated with SMPs; chitosan: shrimp coated with chitosan; SMPs + chitosan: shrimp coated with SMPs and chitosan. The results are the means ± standard deviation. Different lowercase letters indicate significant differences (*p* < .05) between time points in the same treatment, while different capital letters indicate significant differences (*p* < .05) between treatments at the same time point.

### 2.8 Statistical analysis

All experiments were carried out in triplicate, and the results are expressed as mean values. Analysis of variance was performed. The mean comparison was carried out using Duncan's multiple range tests. The data were analyzed using the Statistical Package for the Social Sciences version 19.0 (IBM). Differences were considered significant at *p* < .05.

## 3 RESULTS AND DISCUSSION

### 3.1 pH changes

**FIGURE 2:**
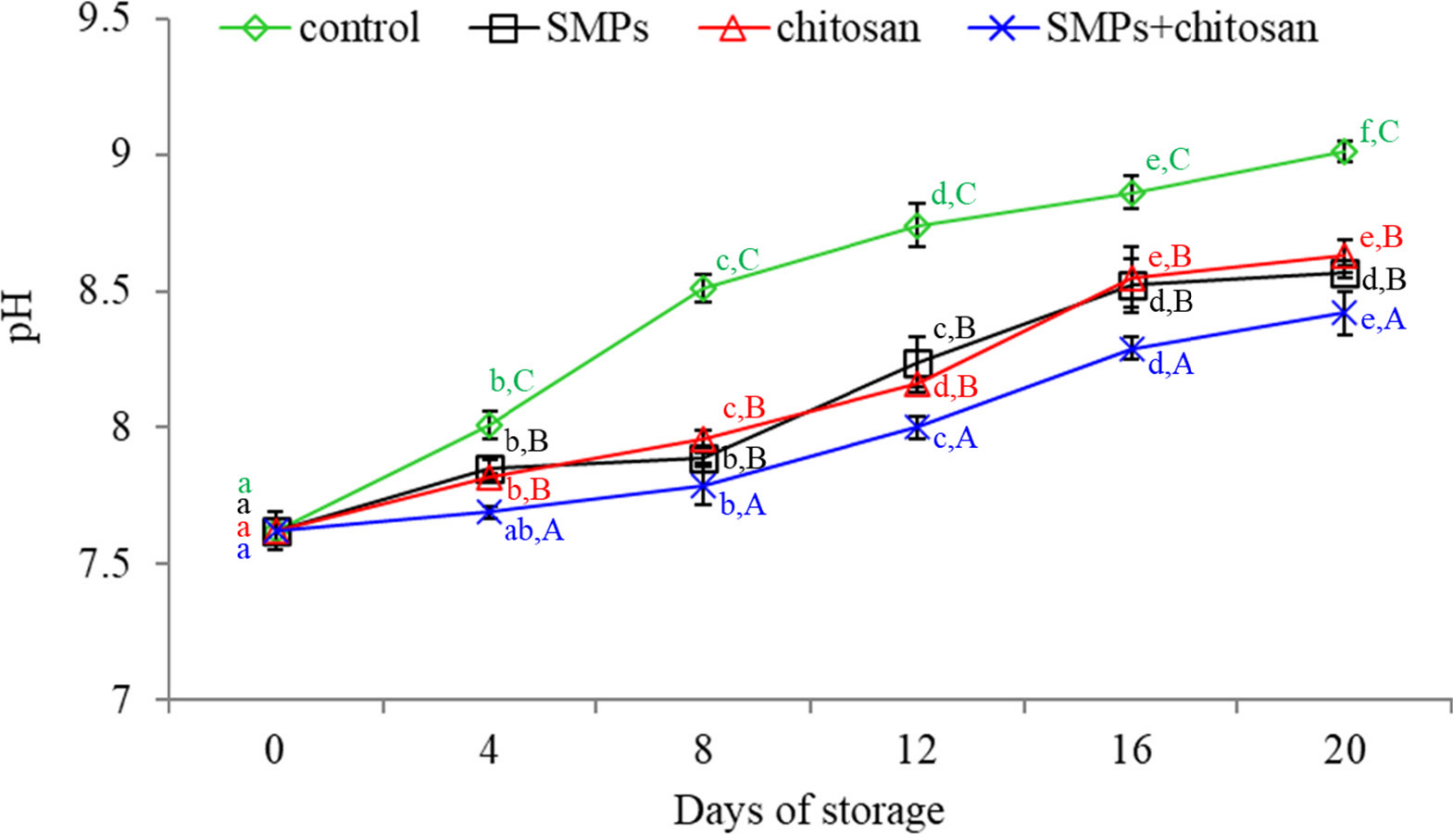
The pH of shrimps during storage at 4 ± 1℃ for 20 days. Control: shrimp without coated; SMPs: shrimp coated with SMPs; chitosan: shrimp coated with chitosan; SMPs + chitosan: shrimp coated with SMPs and chitosan. Bar represents the standard deviation (*n* = 3). Different lowercase letters indicate significant differences (*p* < .05) between time points in the same treatment, while different capital letters indicate significant differences (*p* < .05) between treatments at the same time point

Alterations in pH may indicate the postmortem changes in shrimps and degradation of muscle proteins during long‐term storage (Udayasoorian et al., 2017). Figure 2 illustrates the results of pH changes. The pH of fresh shrimp was 7.62. A constant increase in the pH value was observed in all groups throughout the storage period. However, the highest increase was observed in the control group on all sampling days (*p* < .05). The increase in pH of shrimp was significantly inhibited by the SMPs + chitosan coating, in a manner similar to that reported by Yuan et al. (2016) and Khaledian et al. (2021). After a 20‐day storage period, the pH values of the control, SMPs, chitosan, and SMPs + chitosan groups were 9.01, 8.57, 8.63, and 8.42, respectively. Alparslan et al. (2016) reported that the pH value of shrimp treated with gelatin containing orange leaf essential oil changed from 6.47 to pH 8.57 after storage at 4 ± 1°C for 14 days. The results of the present study are consistent with their findings. Moreover, the pH changes in shrimp are caused by the accumulation of basic compounds due to bacterial activity or enzymatic action (López‐Caballero et al., 2010; Lorenzo et al., 2014). Thus, our present results indicate that SMPs + chitosan may play a pivotal role in maintaining good shrimp quality by lowering the rate of spoilage or decomposition.

In addition, the pH change in the SMPs‐ and chitosan‐coated groups was similar, revealing that SMPs and chitosan effectively maintained the shrimp quality. As the increase of pH in shrimp coated by SMPs + chitosan was significantly lower than SMPs and chitosan coating alone (*p <* .05), there might be a synergistic effect between SMPs and chitosan, but it required further investigation.

### 3.2 Total plate count

**FIGURE 3:**
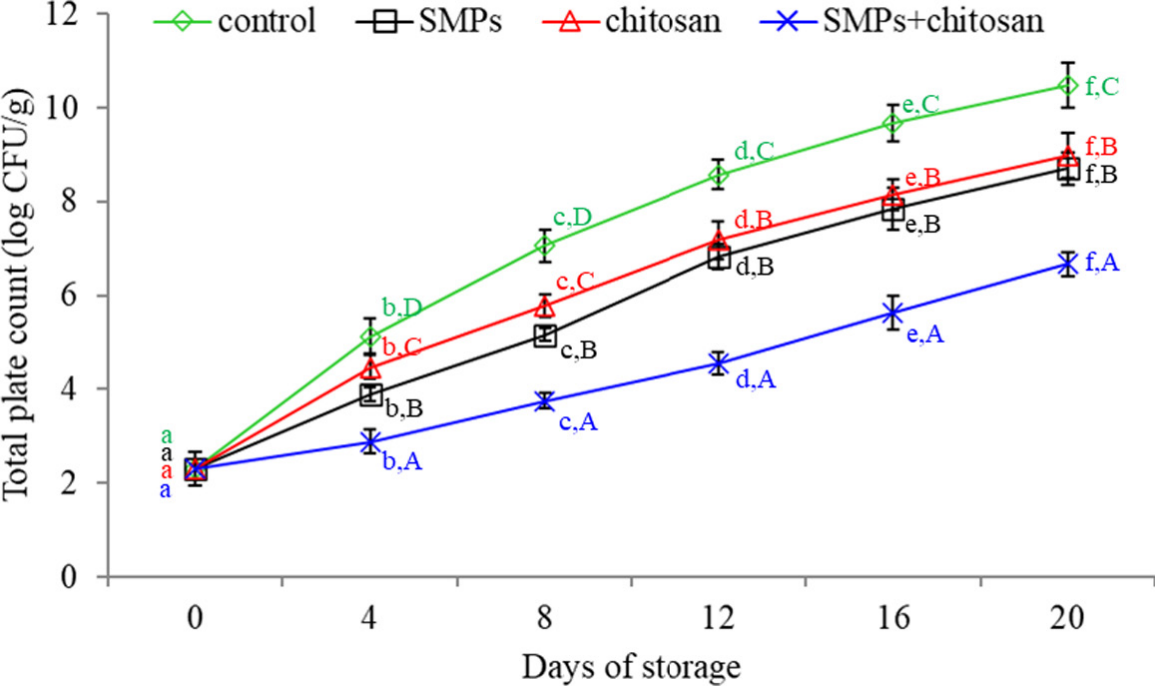
Total plate count of shrimps during storage (4 ± 1℃) for 20 days. Control: shrimp without coated; SMPs: shrimp coated with SMPs; chitosan: shrimp coated with chitosan; SMPs + chitosan: shrimp coated with SMPs and chitosan. Bar represents the standard deviation (*n* = 3). Different lowercase letters indicate significant differences (*p* < .05) between time points in the same treatment, while different capital letters indicate significant differences (*p* < .05) between treatments at the same time point

Figure 3 illustrates the TPC results. The initial TPC of shrimp was 2.31 log CFU/g, indicating that the shrimps used in this study were of good quality. Alparslan et al. (2016) reported similar counts of approximately 2–3 log CFU/g in good quality shrimp. The TPC of shrimps increased with the storage time. The TPC value of fresh crustaceans should not exceed 7 log CFU/g (Udayasoorian et al., 2017). During 20 days of storage, the TPC values of the SMPs + chitosan‐coated group were less than 7 CFU/g, which was deemed acceptable. However, the TPC values of the control, SMPs‐coated, and chitosan‐coated groups were more than 7 log CFU/g after storage for 8, 16, and 12 days, respectively. The low TPC value of the chitosan‐coated group may be explained by the antibacterial activity of chitosan (Hu et al., 2017; Martins et al., 2012; Yuan et al., 2016).

Similarly, the SMPs used in this study could effectively inhibit the growth of various microbes (Dayarian et al., 2014), thereby explaining the low TPC value of the SMPs‐coated group. For the SMPs + chitosan‐coated group, the low TPC value was not only due to the effect of chitosan but also due to the high antibacterial activity of SMPs. The addition of SMPs to chitosan efficiently inhibited microbial growth. There might be a synergistic effect between SMPs and chitosan, but it required further investigation.

### 3.3 PV

**FIGURE 4:**
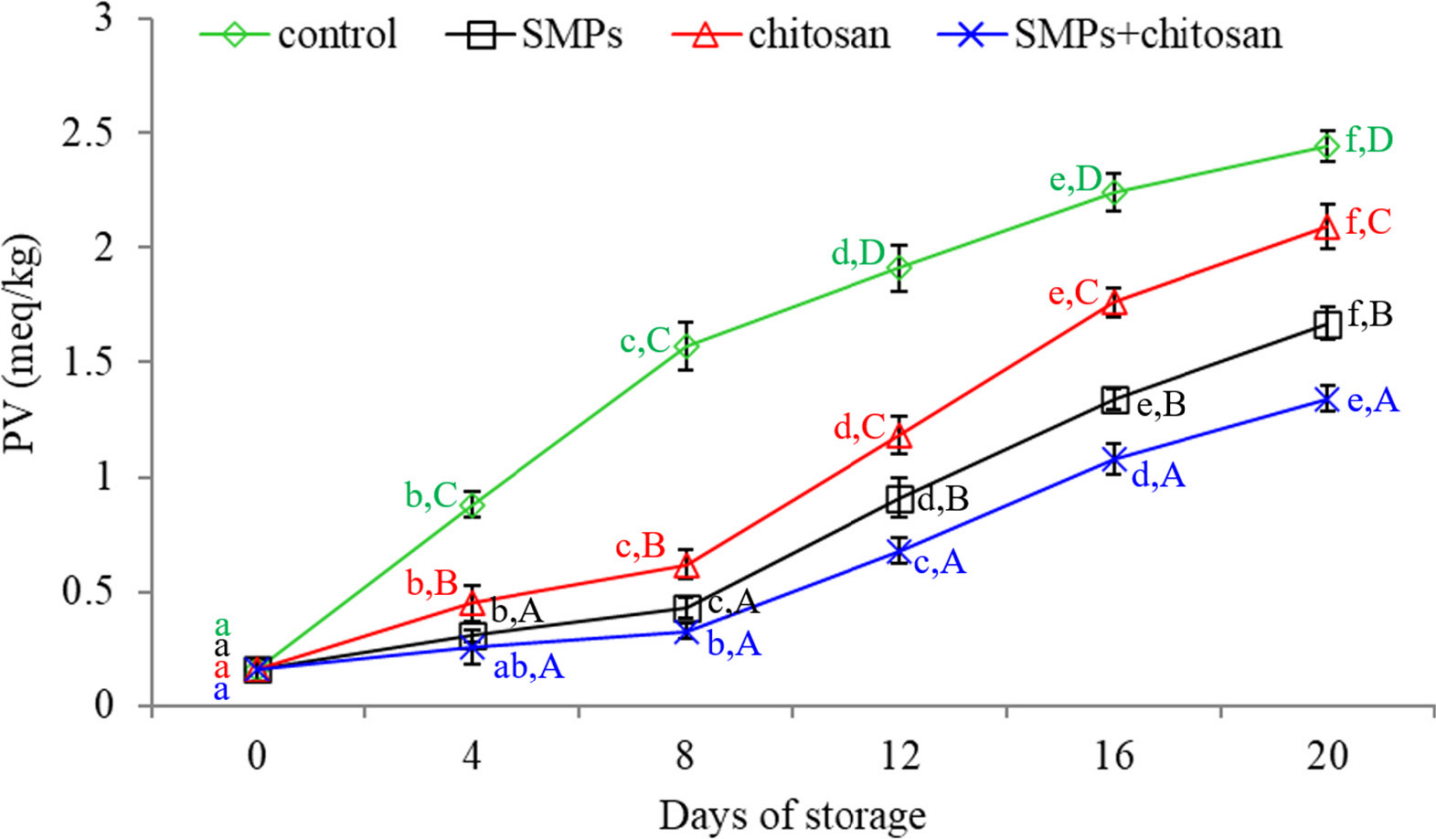
Peroxide value (PV) of shrimps during storage (4 ± 1℃) for 20 days. Control: shrimp without coated; SMPs: shrimp coated with SMPs; chitosan: shrimp coated with chitosan; SMPs + chitosan: shrimp coated with SMPs and chitosan. Bar represents the standard deviation (*n* = 3). Different lowercase letters indicate significant differences (*p* < .05) between time points in the same treatment, while different capital letters indicate significant differences (*p* < .05) between treatments at the same time point

Figure 4 illustrates the results of PV. The PV values continuously increased with storage time. During storage, the control group revealed the highest PV values, followed by the chitosan‐ and SMPs‐coated groups. The PV values in the SMPs + chitosan‐coated group were the lowest. The results of this study were consistent with those of Farajzadeh et al. (2016), who reported that compared to unwrapped samples, shrimps wrapped in a chitosan–gelatin film exhibited lower PV values. At the end of storage, the PV values of the control, SMPs‐coated, chitosan‐coated, and SMPs + chitosan‐coated samples were 2.44, 1.67, 2.09, and 1.34 meq peroxide/kg sample, respectively. The increase in PV values in the SMPs + chitosan‐coated group was lower than that in the other three groups during storage, although the values of all samples were lower than 10 meq/kg of lipid, which is generally regarded as the acceptable level (Jeon et al., 2002).

As shown in Figure 4, the PV values of the SMPs group were significantly lower than those in the chitosan group (Figure 4), thereby indicating that the antioxidant activities of SMPs were higher than those of chitosan. The increase in PV values in shrimp coated with SMPs + chitosan was significantly lower than those of shrimp coated with SMPs and chitosan alone (*p <* .05), indicating that the addition of SMPs to chitosan enhanced the antioxidant activity. There might be a synergistic effect between SMPs and chitosan, but it required further investigation.

### 3.4 TBA index

**FIGURE 5:**
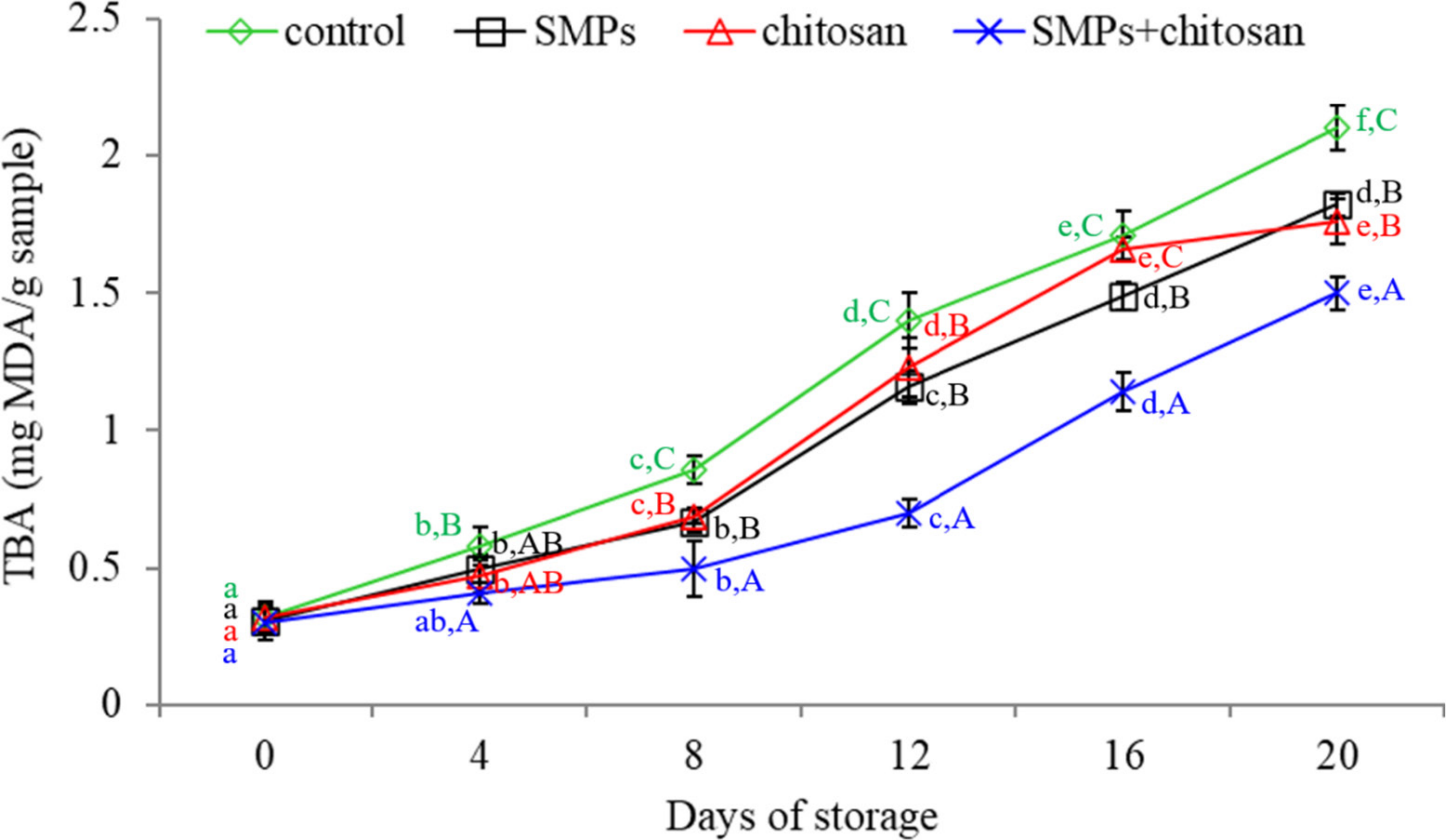
Thiobarbituric acid index (TBA) of shrimps during storage (4 ± 1℃) for 20 days. Control: shrimp without coated; SMPs: shrimp coated with SMPs; chitosan: shrimp coated with chitosan; SMPs + chitosan: shrimp coated with SMPs and chitosan. Bar represents the standard deviation (*n* = 3). Different lowercase letters indicate significant differences (*p* < .05) between time points in the same treatment, while different capital letters indicate significant differences (*p* <.05) between treatments at the same time point

Figure 5 illustrates the TBA results, wherein TBA continuously increases with the storage time. After storage for 20 days, the TBA values of the control group, SMPs‐coated, chitosan‐coated, and SMPs + chitosan‐coated samples were 2.10, 1.82, 1.76, and 1.50 mg MDA/kg, respectively. Peroxide is an unstable compound that causes food odor (Aubourg et al., 2020). MDA, a commonly used indicator of lipid oxidation, is usually measured using the TBA method. Moreover, a high MDA content is associated with rancidity in sensory analyses (Franklin & Mitchell, 2019). According to Farajzadeh et al. (2016), TBA values of 1–2 mg MDA/kg in fish muscle may cause an unpleasant taste and odor. In our study, this value reached the limit for the SMPs + chitosan‐coated group by the 16th day. However, this value was attained by the 12th day in the other groups. Both PV and TBA results indicated that SMPs efficiently inhibited lipid oxidation and thus exerted a protective effect. Indeed, SMPs display high reducing power, strongly scavenge free radicals, and chelate ferrous ions (Jiang et al., 2018). These results indicated that the SMPs + chitosan coating might effectively inhibit lipid oxidation, revealing the potential for use in shrimp preservation. In addition, similar to the trends seen in PV values, the TBA values of shrimp coated with SMPs + chitosan were significantly lower than those of shrimp coated with SMPs alone and chitosan alone (*p* < .05). There might be a synergistic effect between SMPs and chitosan, but it required further investigation.

### 3.5 TVB‐N

Volatile basic nitrogen compounds, which result from the decarboxylation of amino acids following death, act as spoilage indicators (Nirmal & Benjakul, 2011). Thus, the TVB‐N value reflects the degree of protein degradation. Proteins are degraded to amines during storage due to microbial growth, reproduction, and endogenous enzyme activity (Arancibia et al., 2015). The TVB‐N value reflects quality changes in shrimp to a certain extent.

**FIGURE 6:**
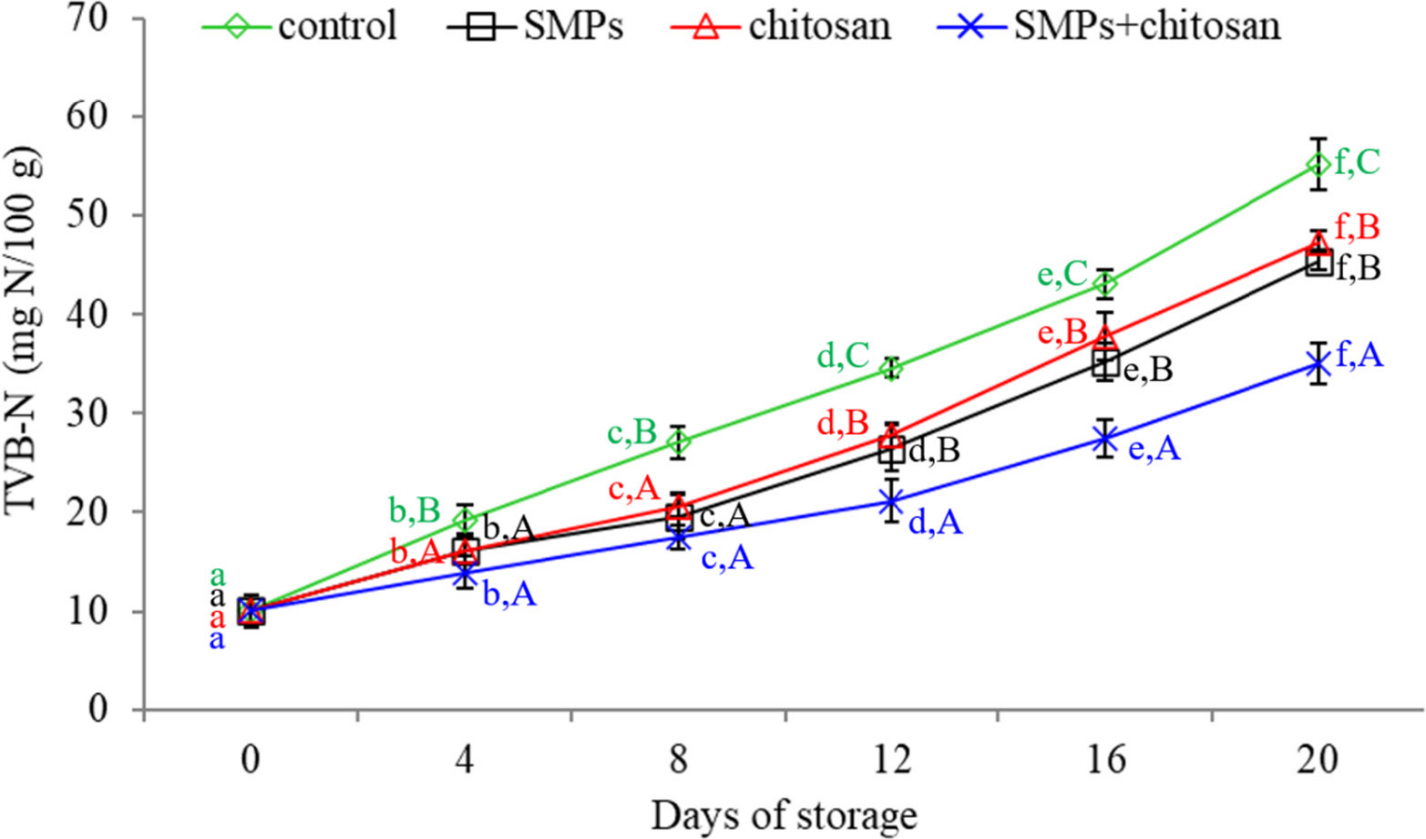
Total volatile basic nitrogen (TVB‐N) of shrimps during storage (4 ± 1℃) for 20 days. Control: shrimp without coated; SMPs: shrimp coated with SMPs; chitosan: shrimp coated with chitosan; SMPs + chitosan: shrimp coated with SMPs and chitosan. Bar represents the standard deviation (*n* = 3). Different lowercase letters indicate significant differences (*p* < .05) between time points in the same treatment, while different capital letters indicate significant differences (*p* < .05) between treatments at the same time point

The initial TVB‐N value of the shrimp was 10.05 mg/100 g (Figure 6), indicating that the shrimp were of good quality (Alparslan et al., 2016; Udayasoorian et al., 2017). During the storage period, the TVB‐N value of all groups increased. However, this increase was slightly moderate in shrimp treated with SMPs or chitosan alone. For marine products, TVB‐N values higher than 30 mg/100 g indicate spoilage (Harpaz et al., 2003). The control (34.59 mg/100 g), SMPs (35.19 mg/100 g), chitosan (37.73 mg/100 g), and SMPs + chitosan (35.04 mg/100 g) groups exceeded the acceptable limit on days 12, 16, 16, and 20 of storage, respectively. Alparslan et al. (2016) reported that the TVB‐N value of shrimp treated with 2% orange leaf essential oil increased by over 30 mg/100 g on day 8. The results of the present study are in accordance with these studies. The TVB‐N value of the SMPs + chitosan‐coated group is the lowest, indicating that the SMPs + chitosan treatment may efficiently inhibit the formation of TVB‐N under storage conditions. In addition, although the TVB‐N values of the SMPs‐coated and chitosan‐coated groups were similar, the increase in TVB‐N values in shrimp coated with SMPs + chitosan was significantly lower than those of the SMPs‐coated and chitosan‐coated groups (*p <* .05). There might be a synergistic effect between SMPs and chitosan, but it required further investigation.

### 3.6 Texture analysis

**FIGURE 7:**
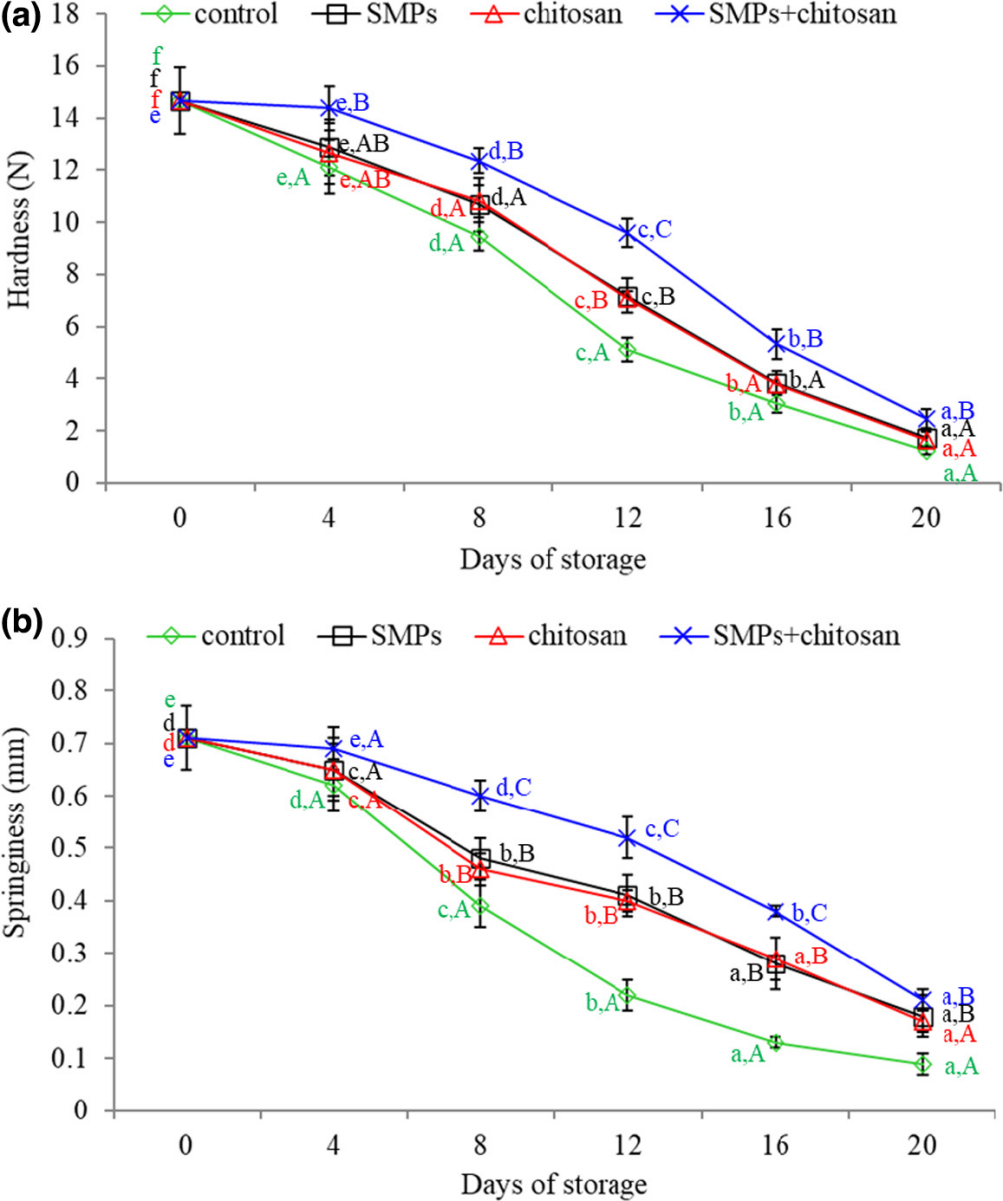
(a) Hardness and (b) springiness of shrimps during storage (4 ± 1℃) for 20 days. Control: shrimp without coated; SMPs: shrimp coated with SMPs; chitosan: shrimp coated with chitosan; SMPs + chitosan: shrimp coated with SMPs and chitosan. Bar represents the standard deviation (*n* = 3). Different lowercase letters indicate significant differences (*p* < .05) between time points in the same treatment, while different capital letters indicate significant differences (*p* < .05) between treatments at the same time point

Texture (hardness and springiness) is crucial in the quality of seafood. Figure 7 illustrates the texture changes in the control and coated shrimp during cold storage. The initial hardness of the shrimp was 14.68 N (Figure 7a). As the storage time increased, the hardness and springiness of shrimps in all the groups decreased significantly (*p* < .05). However, compared to the control group, the hardness (Figure 7a) and springiness (Figure 7b) of shrimps treated with SMPs, chitosan, and SMPs + chitosan under storage conditions were improved. In all the four groups, the highest hardness and springiness values were observed in the SMPs + chitosan‐treated group.

Change in texture parameters is a major factor affecting acceptability. This change is affected by many factors, such as the speed and degree of pH decline after death and the arrangement of muscular protein. The main muscle components leading to texture changes in shrimp are myofibrillar and connective tissue proteins. The results are consistent with that of Wang et al. (2015), who observed that chitosan coating effectively attenuated changes in the texture of shrimp during storage. The improved texture properties of shrimp muscle may be associated with bonding between chitosan and myofibrillar proteins, whereas the final structure is formed via covalent and noncovalent interactions (Yuan et al., 2016).

Furthermore, microbiological processes following seafood death can also lead to the degradation of muscle fibrin and muscle softening. Li et al. (2013) demonstrated that grape seed extract and tea polyphenols could improve the textural parameters of red drum (*Sciaenops ocellatus*). In the present study, the hardness and springiness of shrimp treated with SMPs were significantly improved. The results of the SMPs group were markedly similar to those of the chitosan group, suggesting that SMPs played a pivotal role in inhibiting changes in the texture parameters of shrimp during storage. In addition, the decrease in the texture of shrimp coated with SMPs + chitosan was significantly lower than that of shrimp coated with SMPs alone or chitosan alone (*p <* .05). There might be a synergistic effect between SMPs and chitosan, but it required further investigation.

### 3.7 Sensory analysis

Table 1 summarizes the sensory evaluation results of shrimp subjected to different treatments. All samples revealed a score of nine at the beginning of storage. The sensory scores for both treated and control shrimps declined significantly with increasing storage period. In accordance with the opinions of a group of panelists experienced in shrimp sensory evaluation, the shrimp were considered unacceptable when the sum of characteristic scores decreased below 4. Based on sensory scores, the shelf life for the control, SMPs, and chitosan for the SMPs + chitosan groups was 8, 12, and 16 days, respectively. The decreases in sensory scores in shrimp treated with SMPs, chitosan, and SMPs + chitosan were significantly lower than that of the control shrimp.

Furthermore, the decrease in sensory score in shrimp treated with SMPs + chitosan was significantly lower than those in shrimp treated with SMPs or chitosan alone on 12th and 16th day of storage (*p* < .05), which was consistent with the observations for the pH, TPC, TBA, PV, TVB‐N, and textural parameters. These results suggest that both SMPs and chitosan maintain good shrimp quality. This may be attributed to the high antioxidant and antibacterial activities of SMPs, which reduce lipid oxidation and inhibit microbial growth, thus extending the shelf life and maintaining the quality of shrimp. There might be a synergistic effect between the SMPs and chitosan. Considered together, the results of all the above analyses support the conclusion. But it still required further investigation.

## 4 CONCLUSIONS

The SMPs + chitosan coating suppressed lipid oxidation, inhibited microbial growth, and preserved the texture of shrimp more effectively than the SMPs coating, chitosan coating, or the control. Based on the sensory evaluation under storage conditions at 4 ± 1°C, the shelf life of the untreated samples was found to be 8 days, whereas the shelf life of the samples treated with SMPs + chitosan was prolonged to 16 days. The antioxidant and antibacterial activities of the chitosan coating were enhanced by the incorporation of SMPs. Moreover, there might be a synergistic effect between the SMPs and chitosan, leading to enhanced inhibition of shrimp quality loss. But it still required further investigation. Therefore, SMPs may be used in active packaging materials to maintain the quality of stored shrimp and extend their shelf life.

## CONFLICT OF INTEREST

The authors declare that they do not have any conflict of interest.

## ETHICAL APPROVAL

This study does not involve any human or animal testing.

## Data Availability

The datasets used and/or analysed during the current study are available from the corresponding author on reasonable request.
